# Breakfast characteristics, perception, and reasons of skipping among 8th and 9th-grade students at governmental schools, Jenin governance, West Bank

**DOI:** 10.1186/s40795-021-00451-1

**Published:** 2021-08-06

**Authors:** Manal Badrasawi, Ola Anabtawi, Yaqout Al-Zain

**Affiliations:** Nutrition and Food Technology Department, Faculty of Agriculture and Veterinary Medicine, An-Najah National University, Tulkarm, West Bank PO. Box 7, Nablus, Palestine

**Keywords:** Breakfast, Breakfast skipping, Perception, Palestine

## Abstract

**Background:**

There is growing recognition of the important role of breakfast in children’s nutrition, and the potential harms related to skipping breakfast, including its contribution to obesity and non-communicable diseases. The patterns associated with skipping breakfast may be related to the nutrition transition. This study aimed at exploring the composition of breakfast consumed by Palestinian school children and their perceptions toward this meal. It also aimed at exploring skipping breakfast prevalence, reasons, and its association with selected schoolchildren’s sociodemographic variables and behavioral patterns.

**Methods:**

A cross-sectional online survey was conducted among 12- to 14-year-old schoolchildren from 4 governmental schools in urban and rural areas of the Jenin district in Palestine. The questionnaire included information about children and parents sociodemographic and behaviours, breakfast patterns and composition, reasons for skipping breakfast, and items on schoolchildren’s perception toward breakfast. Statistical analysis of the relevant factors was undertaken using SPSS software.

**Results:**

In a sample of 193 schoolchildren, only 32% reported consuming breakfast all year round. The main reasons reported for skipping breakfast were not feeling hungry, not having the time, and lack of appetite. The vast majority (79%) believed breakfast was beneficial for general health. Sleeping before 10 pm, regular exercise (*p* value < 0.05). and shorter screen time were all significantly associated with a higher level of breakfast consumption (*p* value < 0.01).

**Conclusion:**

Understanding the reasons for missing or skipping breakfast factors which make skipping it more likely, should inform public health strategies to promote breakfast consumption. For example, our findings suggest that awareness of the importance of breakfast was not a significant contributor to skipping breakfast, compared to other structural and cultural factors.

## Introduction

A lot has been researched, written and published about the nutritional transition in low and middle income settings [1]. This transition includes physical activity shifting towards more sedentary lifestyles, and changing dietary habits. In Palestine, such new dietary habits include an increased consumption of saturated fats, reduced fruit and vegetable intake, and reduced fibre intake. They also include an increasing prevalence of skipping breakfast [2]. This transition has been shown to be a significant risk factor for increased non-communicable diseases. Studies show that individuals who skip breakfast are at increased risk of high BMI and type − 2 diabetes [3, 4], which are already at high and increasing rates in Palestine [5, 6]. Globally, skipping breakfast is highest among adolescents, who are consequently at risk of poorer academic performance and physical growth [7]. This makes this issue vitally important in the Palestinian context where one-quarter of the population is made up of adolescents [8].

The most recent comprehensive study studying health behaviours among adolescents was conducted as part of WHO’s Health Behaviour in School-age Children (HBSC) study in 2004 [9]. The study used a cross-sectional survey to investigate health behaviours among adolescents in 35 countries, including the West Bank and Gaza Strip in Palestine. The survey identified several health concerns, including that 45% of adolescents did not consume breakfast, as well as its link to other health behaviours. Several studies since then have considered breakfast consumption among adolescents with similar findings to the HBSC study, in 2009 and 2010 in Palestine, and many other studies in other Arab countries [10–12]. None of these studies, including the HBSC study, considered the underlying reasons for skipping breakfast or people’s perceptions toward it in Palestine.

There are significant and justified concerns about the rising rates of NCDs and obesity in Palestine. Addressing their risk factors necessitates understanding the causes and people’s perceptions of health behaviours which interventions seek to promote [13]. In the case of breakfast skipping, for example, there is a good understanding of the problem, but not of its causes or people’s perceptions toward breakfast meal. Therefore, a deep and compressive understanding of skipping breakfast helps come out with appropriate interventions seeking to promote breakfast as a positive health behaviour. The aim of this study is, to explore breakfast consumption in a representative sample of children aged 12–14 years. The objectives are first to understand the associations of skipping breakfast with age, gender, parental education level, and sleeping time, as potential determinants of skipping breakfast. Second, to explore the associations between the reasons for skipping breakfast and perceptions towards breakfast consumption. Third, to investigate breakfast characteristics in association with the previous objectives.

### The methodology and study instruments

This study used a cross-sectional design to answer the research objectives. The participants were grades eight and nine schoolchildren selected from four governmental schools in Jenin District in the West Bank, Palestine. The four schools included two girls’ and two boys’ schools, randomly selected from urban and rural areas. A formal letter was sent to the Ministry of Education to get permission for the data collection from the selected schools.

### Data collection

Data collection started in October 2020 and ended in November 2020. The final version of the questionnaire described above was formulated into an online survey. The first step of the data collection was schools selection; 4 schools from urban and rural areas of Jenin governance were invited by the Ministry of Education. The distribution of the link for the online data sheet started after the verbal consent of participation was taken from the school principals and the parents of schoolchildren. The students were invited to fill in the online forms through their teachers by sending the link through the e-classes and through the schools’ webpages. The students were briefed on how to fill out the online questionnaire by their teachers and they were asked to sign the consent form of participation online. The students were asked to report the correct data regarding their personal information and they were told to answer the questions individually. They were also informed that there were no wrong or correct answers and the data would be used only for research purposes.

### Ethical approval

The Palestinian Ministry of Education approved the study protocol once it had been reviewed by the Research and Quality Control committee. The study protocol was also approved by the Institution Review Board (IRB) ethical committee at An-Najah National University. Having got the approval, an invitation letter was sent to the selected schools through the Directorate of Education- Jenin office. As the participant’s age was less than 18 years old, the consent of participation was taken first from the school principals, then from the parents through the school teachers. Informed consent was obtained from all the parents before the data collection. The online questionnaire was sent to the students through their teachers in the selected schools after the teachers got the parents’ agreement. All students were informed that their participation was not compulsory and the data would be used only for research purposes. They were asked to answer the questions individually.

### Sample size and sampling procedures

Using G power software and an alpha of 0.05 (two-sided) and 80% power, sample size calculations revealed that a minimum of 175 participants were required to determine the prevalence of breakfast skipping. Then the sample size was calculated again to determine the association between breakfast skipping and other variables. The mean difference test was selected, 5% level of significance, (80%) power. The required sample size was 208 participants. The inclusion criteria were: all students who are in grades 8th and 9th from Jenin governmental schools in Jenin are willing to participate and have completed the online questionnaire. The students, who did not answer the primary questions i.e. questions related to breakfast, reasons for skipping and perceptions toward breakfast, were excluded from the final data analysis. A total of 1250 students from grades 8th and 9th from 4 different schools were invited to fill in the online questionnaire. Only 203 students responded to the invitation and gave the required data, with a 16% responding rate. Ten (10) students were excluded due to missing data; and only 193 students were included in the final analysis.

### Research instrument

The questionnaire was developed based on a thorough literature review pertaining to breakfast consumption among school students from different age groups. Two specialists in nutrition prepared the initial questionnaire items using the participants’ native language (i.e. Arabic). Content validity was examined by five (5) experts in nutrition and three (3) in assessment and research methods. A few items were amended based on the experts’ comments and suggestions. Other items were deleted and replaced with new ones (i.e. items related to reasons for breakfast skipping, mainly the repeated or unclear ones) to ensure that all items measure the investigated construct accurately. The revised questionnaire was sent for Arabic language editing before it was distributed. The final version of the questionnaire consisted of three sections: section one was for socio demographic characteristics of the students, including gender, area of living, family type, number of family members, parents’ education, parents’ work status and pocket money. The second section included the medical history, lifestyle and presence of chronic diseases. The lifestyle variables included sleeping hours, sleeping time, wake up time, methods of going to school (walking or using transport), going for exercise and screen time. The third section had breakfast related data: 3 items about breakfast consumption during school and weekend days (answers: always if students eat breakfast 6–7 days/week, sometimes if students eat breakfast less than 5 times or less per week, never if students do not eat breakfast at all) in addition to consuming meals at schools, two open-ended questions about the types of food consumed in breakfast and school meals, eight dichotomous questions with three point Likert scale: agree, no opinion and disagree. The reliability test was done using Chronbach alpha test for each section separately. The reliability for the reasons for skipping breakfast was 0.81, and the reliability for perceptions was 0.59 for the seven items; however, the reliability for the six items was improved by deleting item no. 7.

### Statistical analysis

The Statistical package for the social Sciences SPSS, version 21, was used to analyze the collected data. Descriptive analysis including means and standard deviations were used to analyze data pertaining to continuous dependent and independent variables. The categorical data was described by percentages. Independent t-test and ANOVA tests were conducted to examine the differences between selected independent variables, while the Chi Square test was employed to examine the association between the categorical independent variables and the nominal levels, with a significance level of 0.05.

## Results

### Characteristics of the participants

A total of 193 students were included in the final analysis. The majority of the sample are girls (70.5%), eighth grade (57%), and live in a nuclear family (88.1%). The number of family members ranged from 3 to 15 members; 18.2% of the families have ≤5 members, 70.% have (6–8 members) and 11.5% of the family members are 9 and above. The means of pocket money as reported by the participants is 5.4 ± 3.2, NIS-New Israeli Shekel−/day. In regard to lifestyle, (83.7%) of the students walk to school every day and only (19.3%) go for exercise regularly. The mean of screen time is 4.4 ± 2.8 h/day. The mean of sleeping hours is 8.1 ± 1.4, hour/day.

### Prevalence of breakfast consumption and meal characteristics

**Fig. 1:**
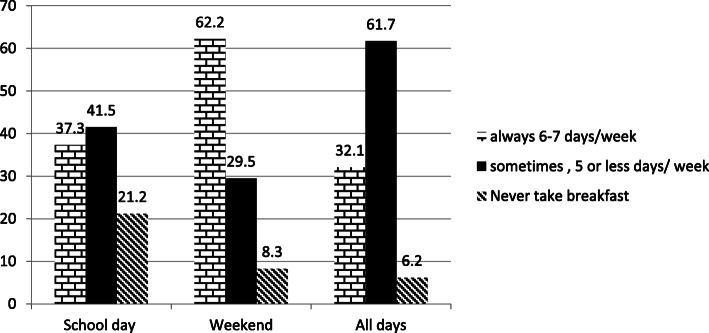
Breakfast and school meal consumption during school and weekend days

Figure 1 shows the prevalence of breakfast consumption on school days, weekends and all days. The majority of participants (62.2%) reported eating breakfast on weekends, followed by school days (37.3%) and all days (32.1%). While the prevalence of breakfast skipping was higher in school days as compared to weekends. In regard to taking meals to school, 80.3% of the students said they take meals to school every day, while the rest said they do not take meals to school.

### Breakfast consumption and associated factors (socio-demographic and lifestyle)

Table 1 shows the association between breakfast consumption with sociodemographic variables and lifestyle. Using the chi square test, the results show that there is no relationship between breakfast consumption in general, schooldays, or on weekends, gender, grade, family type, and parents ‘education, or parents’ work status. However, the every day breakfast consumption is associated with living in cities; and never consuming breakfast is associated with living in villages, *p* < 0.05 using the Chi Square test. Moreover, the relationships between breakfast consumption with the number of family members and the amount of pocket money are not significant using one way ANOVA test. In regard to lifestyle factors, early sleeping i.e., before 10:00 pm is significantly associated with a higher prevalence of breakfast consumption, *p* < 0.05, using the Chi Square test. Similarly, a significant association is found between going for exercise (regular, irregular) with a higher prevalence of every day breakfast consumption, *p* < 0.05, using the Chi Square test, whereas the other lifestyle variables (walking to school, waking up early) show a non-significant association with breakfast consumption. For continuous variables in the lifestyle (i.e. hours of night sleeping and hours of screen time), the results show a significant relationship between breakfast consumption and screen time; students who eat breakfast every day reported shorter screen time (3.3 ± 2.3) hours/ day, students who sometimes eat breakfast (4.66 ± 3.1) hours compared to who always skip breakfast (5.4 ± 2.7) hours/ day, *p* < 0.01 using one way ANOVA test. Similar trend is shown for hours of night sleep; students who skip their breakfast reported shorter night sleep (7.2 ± 2) compared to students who always or sometimes eat breakfast with (8.1 ± 1.1 and 8.1 ± 1.3) respectively, with no significant differences using one way ANOVA test, *p* > 0.05.

**Table 1:** Participants’ breakfast consumption according to sociodemographics, medical history and lifestyle characteristics presented in n (%)/ mean ± sd

	Total N (%)	Breakfast consumption			*P* value
		All days	Sometimes	Never	
**Socio demographic**					
Gender					
Boys	57 (29.5)	18 (31.6)	32 (56.1)	7 (12.3)	0.527
Girl	136 (70.5)	44 (32.4)	67 (49.3)	25 (18.4	
School location					
City	103 (53.4)	40 (38.8)	51 (49.5)	12 (11.7)	0.039 *^1^
Village	90 (46.6)	22 (24.4)	48 (53.3)	20 (22.2)	
Grade					
8th	110 (57)	40 (36.4)	55 (59)	15 (13.6)	0.24
9th	83 (43)	22 (26.5)	44 (53)	17 (20.5)	
Family type					
Nuclear family	170 (88.1)	52 (30.6)	89 (52.4)	29 (17.1)	0.459
Extended family	23 (11.9)	10 (43.5)	10 (43.5)	3 (13)	
Parents’ education					
Both parents have primary education	20 (10.4)	4 (20)	14 (70)	2 (10)	0.064
Both parents have secondary education	31 (16.1)	15 (48.4)	(11 (35.5)	5 (16.1)	
Both parents have university degree	40 (20.7)	12 (30)	23 57.5)	5 (12.5)	
Mixed level; primary /secondary	34 (17.6)	16 (47.1)	14 (41.2)	4 (11.8)	
Mixed level; primary /university degree	43 (22.3)	10 (23.3)	25 (58.1)	5 (18.6)	
Mixed level; secondary /university degree	25 (13)	5 (20)	12 (48)	8 (32)	
Parents working status					
Both parents are not working	5 (2.6)	2 (40)	3 (60)	0 (0)	0.579
Both parents are working	47 (24.4)	12 (25.5)	28 (59.6)	7 (14.9)	
One of parents’ work	140 (72.5)	48 (34)	68 (48.2)	25 (17.7)	
**Medical history**					
Presence of Chronic diseases					
Yes	19 (9.8)	8 (42.1)	8 (42.1)	3 (15.8)	0.604
No	174 (90.2)	54 (31)	91 (52.3)	29 (16.7)	
History of surgical operation					
Yes	8 (4.1)	4 (50)	3 (37.5)	1 (12.5)	0.541
No	185 (95.9)	58 (31.4)	96 (51.9)	31 (16.8)	
**Lifestyle**					
Go to school					
Walking	159 (83.7)	48 (30.2)	83 (52.2)	28 (17.6)	0.335
Using transports (public or private)	31 (16.3)	13 (41.9)	15 (48.4)	3 (9.7)	
Go for exercise (home, outside)					
Yes regularly	37 (19.3)	17 (45.9)	17 (45.9)	3 (8.1)	0.015
Yes, irregular	109 (56.8)	35 (32.1)	59 (54.1)	15 (13.8)	
No	46 (24)	9 (19.6)	23 (50)	14 (30.4)	
Wake up time					
Before 6:00 am	24 (12.9)	8 (33.3)	9 (37.5)	7 (29.2)	0.09
6:00–7:00 am	98 (52.7)	37 (37.8)	49 (50)	12 (12.2)	
7:00–8:00 am	47 (25.3)	8 (17)	31 (66)	8 (17)	
After 8:00 am	17 (9.1)	6 (35.3)	9 (52.9)	2 (11.8)	
Sleep time					
Before 9:00 pm	21 (11.4)	5 (23.8)	14 (66.7)	2 (9.5)	
9:00–10:00 pm	70 (37.8)	31 (44.3)	36 (51.4)	3 (4.3)	0.002**^1^
10:00–11:00 pm	36 (19.5)	10 (27.8)	20 (55.6)	6 (16.7)	
After 11:00 pm	58 (31.4)	13 (22.4)	28 (48.3)	17 (29.3)	
Continuous variables (presented in mean ± sd)					
	All the time (mean ± sd)	Sometimes (mean ± sd)	Never (mean ± sd)	P value	
Number of family members	6.7 ± 1.5	6.9 ± 1.7	6.5 ± 1.4	0.526	
Pocket money (NIS/ day)	5.6 ± 4.3	4.9 ± 2.1	6.3 ± 3.7	0.063	
Sleeping hours	8.1 ± 1.1	8.1 ± 1.4	7.5 ± 1.7	0.061	
Screen time	3.3 ± 2.3	4.7 ± 3.1	5.4 ± 2.7	0.002**^2^	

*Significant at p < 0.05, ** significant p < 0.01

^1^ using chi square test, ^2^ using one way ANOVA

### Breakfast meal characteristics

Table 2 shows the breakfast and school meal components. Sandwiches are the most consumed type of meal in both breakfast and school meals. Breakfast is mostly prepared by the parents; and students eat their breakfast with their siblings (41.1%), and with their whole family (34.4%).

**Table 2:** Breakfast and school meal characteristics and consumption pattern presented in n (%)

	n	%
**Breakfast characteristics (school days)**		
Type of food		
Sandwich (cheese, labaneh, Zaater, sausage)	80	53
Milk+ cookeies (cake +biscuits)	17	11.3
Tea +cookies (cake +biscuits)	10	6.6
Milk+ cereals	12	7.9
Traditional breakfast	32	21.2
Consumption company		
Alone	37	24.5
With siblings	62	41.1
With family	52	34.4
Breakfast preparation		
Parents / mothers	113	74.8
Ready food from outside the house	6	4.0
I prepare it myself	28	18.5
Brothers or sisters	4	2.6
**School meal characteristics**		
Type of food		
Sandwich (Cheese, labaneh, chocolate)	89	74.7
Sandwich + fruits	5	4.2
Sandwich and juices	9	7.6
Cake or cookies, chips	6	5
Fruits only	3	2.5
Sandwich+ cookies or chocolate or cake	7	5.9

### Breakfast skipping reasons and associated factors

Table 3 shows the reasons for breakfast skipping as reported by 129 students who always or sometimes skip breakfast. The most common reason for skipping breakfast is that “*they don’t feel hungry in the morning*,” followed by “*they don’t have time to eat*” and *“they don’t like to eat early*,” while “*they don’t like the food*” and “*they want to lose weight*” are the least common. The results reveal that there is an association between female gender and the eight reasons for breakfast skipping, *p* > 0.05 using the Chi Square test. The same finding is found with school location, grade, students living area and family type. For parents’ education, there is a significant association between item 5 (*I don’t find ready food to eat*) with lower parents’ education level, *p* < 0.05 using the chi square test. The same significant association was found with parents’ working status (both parents are not working), *p* < 0.05.Likewise, item.7 (*My family skips breakfast and so I do*), is associated with both parents are not working, *p* < 0.01 using the Chi square test.

**Table 3:** Students (Yes) answer for reasons for breakfast skipping

Reasons	n	%
I don’t feel hungry	77	59.7
No time to have breakfast	65	50.4
I don’t like to eat early	63	48.8
I feel uncomfortable when I eat	42	32.6
I don’t find ready food to eat	25	19.4
I need to lose weight	20	15.5
My family skip the breakfast and so I do	30	23.3
I don’t like the food choices	19	14.7

### Perceptions toward breakfast and associated factors

As shown in Table 4, students reported different perceptions toward breakfast. Breakfast consumption is very important for general health (79.3%) and for good cognitive performance (81.3%), while 64% disagree that it increases weight, (69.9%) disagree that it causes gastrointestinal disturbances, and 81.3% disagree that it makes them feel lazy and less energetic. No significant relationship was found between students’ perceptions and any of the socio demographic variables: gender, school locations, parents’ education or parents’ working status, *p* > 0.05.

**Table 4:** Students’ positive and negative perceptions towards breakfast consumption

Perceptions	n	%
I believe breakfast consumption is very important to general health		
Agree	153	79.3
No opinion	20	10.4
Disagree	20	10.4
I believe breakfast consumption increase the concentration and memorization during the classes		
Agree	157	81.3
No opinion	19	9.8
Disagree	17	8.8
I believe breakfast consumption increase the weight		
Agree	25	13.0
No opinion	44	22.8
Disagree	124	64.2
I believe breakfast consumption may leads to gastrointestinal disturbances		
Agree	13	6.7
No opinion	45	23.3
Disagree	135	69.9
I believe breakfast consumption make me feel lazy and less energetic		
Agree	19	9.8
No opinion	17	8.8
Disagree	157	81.3
I believe I can make up the breakfast with other meals during the day without any difference on health		
Agree	57	29.5
No opinion	33	17.1
Disagree	103	53.4

## Discussion

Findings of this study indicate that only 32% of children consume breakfast daily all-year round, and 62% consume breakfast daily during the weekend. Previous studies with similar samples conducted in Palestine reported 50% breakfast consumption twice or fewer per week [10], 45% breakfast consumption 4 days-per-week during school term [14], and 62% breakfast consumption [11]. The latest study with a similar sample from a neighbouring country, Jordan reported 80% of children consuming breakfast [15]. In Saudi Arabia, a survey recently reported that 79% of children skipped daily breakfast but that there was a higher breakfast consumption at weekends compared to weekdays [12]. Our findings indicate a lower proportion of children consuming breakfast in Palestine than in neighbouring countries.

Breakfast consumption was not associated with gender, age, parents’ education, parents’ work status, or pocket money, but was positively associated with living in a village. Breakfast consumption was significantly associated with regular exercise, sleeping before 10 pm, and shorter screen time. Previous studies in the same region found different associations. For example, an association between female gender and breakfast consumption has been observed [10, 11, 16]. However, this association is not seen in Saudi Arabia or Jordan [12, 15]. A relationship between education and breakfast consumption has also been seen, with a higher parental education level predicting lower breakfast skipping [10, 12], and a higher paternal level of education predicting lower breakfast skipping [14]. In 2009, no relationship was seen between rural or urban living and breakfast consumption [11]. The same study also found an association between breakfast consumption and socioeconomic status.

Almost 70% of children in the current study had more than 2 h of screen time per day, with a higher proportion among children, and 66% did not sleep the recommended amount, both of which were associated with reduced breakfast consumption [17]. Other studies have found similar associations, with waking up late associated with skipping breakfast [18], and longer sleep associated with decreased breakfast skipping [19].

Previous surveys on a regional level have not studied the relationship between breakfast consumption and exercise. Regular breakfast consumption was associated with health-promoting behaviours including exercise [20], and adolescents who skip breakfast are less likely to exercise regularly [21].

Of those consuming breakfast at home, 35% ate sandwiches (white cheese, *labaneh*, *zaatar*), 8% ate cereal and milk, and 18% cookies with milk or tea. 40% had breakfast prepared by their parents. A study conducted in Qatar, surveying a younger age group, found that 90% ate eggs or cheese for breakfast, and 42% sweets and chocolate [22]. In a study in Saudi Arabia, 48% had fried eggs sandwiches, 46% had breakfast cereal, and 41% had spread cheese sandwiches. In the same sample, 80% had breakfast prepared by parents, and only 4–7% consumed more healthy options (such as *labaneh* or *zaatar* sandwiches) [17].

### Reasons for skipping breakfast

In our sample, the most common reasons given for skipping breakfast were not feeling hungry (60%), not having time (50%), and not enjoying eating early (49%). Other reasons included a desire to lose weight (15%) and the rest of the family skips (23%).There was a statistically significant association between being female and all the aforementioned factors. Both parents being out of work and lower education level were significantly associated with children reporting not having anything to eat (20%). These findings are similar to reasons identified in other studies in Arab countries and globally. In a study in Jordan, the top reasons were poor appetite (65%), not having time (60%), having nothing to eat (60%), and having no one to prepare breakfast (58%) [15]. In Saudi Arabia, 48% reported not feeling hungry and 36% reported not having time [17]. In an Australian study, the two most common reasons were a lack of hunger and a lack of time [23].

### Perceptions for breakfast

There were generally positive perceptions of breakfast among our sample, with 79% believing it is beneficial for general health, 81% agreeing that it increases concentration and memorisation at school, only 13% believing that it contributes to weight gain, and 81% disagreeing that breakfast consumption made them lazy and less energetic. Such perceptions have not been found in previous research in the Arab world. There have been studies investigating perceptions of breakfast elsewhere in the world, but with very different samples and methodologies [24–26].

### Implications

Many studies have investigated patterns of breakfast consumption and its associations with various individual and societal factors. This study has delved deeper into the reasons behind skipping breakfast, and children’s perceptions toward breakfast. This study examined factors which have not previously been investigated in Palestine, such as the relationship of children’s breakfast consumption with exercise, sleep, and screen time. These associations are particularly important because they form part of the nutritional transition.

The current research findings have led to important public health implications. For example, perception of breakfast is generally positive, with a high level of awareness of its importance and role in improving performance at school and health in general. Public health campaigns educating children on the importance of breakfast are therefore unlikely to be valuable. For a small subset of children, particularly girls, perception of weight may, on the other hand, be an important factor to address. This is particularly important in light of the increasing rates of eating disorders in Palestine.

It is essential to note that, currently in Palestine, there are no policies in place to mandate providing breakfast at schools for any age group in any class. There are only strategies and policies to control the type of food supplied into school canteens. This includes banning specific high-fat processed foods (i.e. chips and crisps). On a local level, there are some private initiatives to provide home-made food to schools.

The lack of association between breakfast consumption and parents’ work status was a surprising finding, as higher education and work-status are normally associated with increased healthy behaviour and have been found to be positively associated with breakfast consumption in previous studies in Palestine and neighbouring countries. Yet, important reasons for skipping breakfast included having no one to prepare breakfast and skipping breakfast because the rest of the family does. It is vital to understand the context defining public health issues to facilitate the design and implementation of effective public health interventions. In relation to breakfast consumption, for example, it is important to appreciate the nutrition transition, and it’s various components, such as sedentary lifestyles, increased focus on weight, higher exposure to technology, and higher female employment rates. One potential intervention to improve breakfast consumption in Palestine may be to promote breakfast provision in schools. However, this would need a more comprehensive exploration of the characteristics of breakfast for different age groups and backgrounds. This was touched upon in our survey but would need more detailed investigation. In addition, the focus should be on further examining the reasons for skipping breakfast, nutritional education to eliminate misperceptions about breakfast, and correction of unsuitable nutritional components of breakfast.

## Strengths and limitations

### Strengths

This study sheds light on factors related to breakfast consumption which are vital to designing public health interventions. These include a detailed investigation of the different associations between breakfast consumption and different characteristics of children and their parents, as well as factors which have not been looked at in the Palestinian context, such as exercise, sleep, and screen time. The survey also offered valuable insight into perceptions of breakfast and reasons for skipping it. In addition, the sample we surveyed is representative of the selected studied age group population.

### Limitations

There was a relatively low response rate to the survey, which may challenge the generalisability of the findings, despite the representative sample. Although it is uncommon for studies with the same population in the neighboring countries to have this low response rate, [15, 27] it is important to consider that the main reason for this low rate might be using online survey due to the covid-19 pandemic, which has made it difficult meet school children face to face. Within the survey, there was possible overlap in the definition of breakfast between the meal consumed at home before school, and the one consumed at school in the morning. This may have had an impact both on the responses and their analysis. Finally, although we collected basic information on the characteristics of breakfast which children consumed, this was an open-ended qualitative question. This means that there was insufficient detail to quantify what children consumed.

## Conclusion

Breakfast is well-recognised as an important meal in our sample of Palestinian 8th and 9th school children. However, these schoolchildren are not consuming breakfast consistently due to several reasons or obstacles. On an individual level, these include long screen time and short sleep. On a family level, household factors, such as not having anyone to prepare food, and perceptions or fears around weight are important determinants. Public health interventions would therefore have higher chances of success if targeted at addressing such factors, rather than simply promoting breakfast. Especially that students of this sample have shown good or positive perception towards breakfast consumption.

Future research should aim to better understand the culture around breakfast, such as what children eat, and where they would be prepared to eat it, to support the design of effective public health interventions around it.

## Data Availability

The dataset used and analysed in this study is available from the corresponding author on reasonable request.
